# Pattern-Triggered Immunity Suppresses Programmed Cell Death Triggered by Fumonisin B1

**DOI:** 10.1371/journal.pone.0060769

**Published:** 2013-04-01

**Authors:** Daisuke Igarashi, Gerit Bethke, Yuan Xu, Kenichi Tsuda, Jane Glazebrook, Fumiaki Katagiri

**Affiliations:** 1 Department of Plant Biology, Microbial and Plant Genomics Institute, University of Minnesota, St. Paul, Minnesota, United States of America; 2 Institute for Innovation, Ajinomoto Co., Inc., Kawasaki-ku, Kawasaki, Japan; Ghent University, Belgium

## Abstract

Programmed cell death (PCD) is a crucial process for plant innate immunity and development. In plant innate immunity, PCD is believed to prevent the spread of pathogens from the infection site. Although proper control of PCD is important for plant fitness, we have limited understanding of the molecular mechanisms regulating plant PCD. Plant innate immunity triggered by recognition of effectors (effector-triggered immunity, ETI) is often associated with PCD. However pattern-triggered immunity (PTI), which is triggered by recognition of elicitors called microbe-associated molecular patterns (MAMPs), is not. Therefore we hypothesized that PTI might suppress PCD. Here we report that PCD triggered by the mycotoxin fumonisin B1 (FB1) can be suppressed by PTI in Arabidopsis. FB1-triggered cell death was suppressed by treatment with the MAMPs flg22 (a part of bacterial flagellin) or elf18 (a part of the bacterial elongation factor EF-Tu) but not chitin (a component of fungal cell walls). Although plant hormone signaling is associated with PCD and PTI, both FB1-triggered cell death and suppression of cell death by flg22 treatment were still observed in mutants deficient in jasmonic acid (JA), ethylene (ET) and salicylic acid (SA) signaling. The MAP kinases MPK3 and MPK6 are transiently activated and inactivated within one hour during PTI. We found that FB1 activated MPK3 and MPK6 about 36–48 hours after treatment. Interestingly, this late activation was attenuated by flg22 treatment. These results suggest that PTI suppression of FB1-triggered cell death may involve suppression of MPK3/MPK6 signaling but does not require JA/ET/SA signaling.

## Introduction

Programmed cell death (PCD) is a genetic program for cellular suicide and plays an important role during immunity, biotic stress responses, development and senescence in multicellular organisms [1]. The hypersensitive response (HR) to pathogen infection is a type of plant PCD that has been extensively studied [1]. Although the biological relevance of HR-PCD is not fully understood, its timely activation and suppression are thought to be important for plant fitness.

Pattern-triggered immunity (PTI) is one type of plant innate immunity that is triggered upon the perception of microbe-associated molecular patterns (MAMPs) by the cognate pattern recognition receptors (PRRs) [2]. *Arabidopsis thaliana* recognizes a variety of MAMPs, including flg22 and elf18, peptides originated from bacterial flagellin and EF-Tu, respectively, and fungal chitin [3], [4], [5], [6]. Flg22, elf18, or chitin treatment leads to enhanced resistance to pathogens, showing that PTI contributes to resistance [6], [7], [8], [9].

Analysis of an Arabidopsis *dde2*/*ein2*/*pad4*/*sid2* quadruple mutant showed that jasmonic acid (JA), ethylene (ET), and salicylic acid (SA) signaling all contribute positively to flg22-triggered immunity measured as growth inhibition of a virulent bacterial strain, *Pseudomonas syringae* pv. *tomato* DC3000 (*Pto* DC3000), in leaves [9]. DDE2, EIN2 and SID2 are essential components of JA, ET, and SA signaling, respectively [10], [11], [12]. The *pad4* mutation affects SA signaling and other immune signaling [13], [14]. Although flg22-triggered immunity is only weakly affected in plants with mutations in any one of these genes, it was mostly abolished in the quadruple mutant [9].

PTI apparently suppresses HR triggered by inoculation of ETI-triggering bacterial strains [15], [16], [17], [18]. Overlapping infiltration assays showed that HR triggered by avirulent bacterial pathogens was typically inhibited in the overlapping region that was pretreated with flg22 or the non-host pathogen, PTI-inducing *Pseudomonas fluorescens* [17]. This suppression of HR by PTI is due to restriction of the type III protein secretion system’s ability to inject type III effectors [18]. It is host recognition of these effectors that triggers HR. Thus, it remains unknown whether PTI interferes with the plant signaling mechanisms that lead to induction of the HR.

Fumonisin B1 (FB1) is a mycotoxin produced by several species of Fusarium molds, including *Fusarium verticillioides*, which elicits PCD in both plant and animal cells [19], [20], [21]. FB1 treated leaves develop lesions with characteristics reminiscent of HR, including the generation of reactive oxygen species, deposition of phenolic compounds and callose, accumulation of camalexin, and expression changes of defense-related genes [21]. An Arabidopsis mutant deficient in vacuolar processing enzymes (VPEs), which are vacuole-localized cysteine proteases, is insensitive to FB1, indicating that VPEs are essential for FB1-triggered cell death [22].

Perception of flg22 triggers the activation of a mitogen activated protein kinase (MAPK) cascade consisting of MKK4 and MKK5 [mitogen-activated protein kinase kinase (MAPKK)] and MPK3 and MPK6 (MAPK) [23]. MPK3 and MPK6 are also activated by other MAMPs such as elf18 and chitin [5], [8]. Activation of the endogenous MAPKs by induced expression of a constitutively active form of MKK4 or MKK5 leads to HR-like cell death [24]. An *mpk6* T-DNA insertion mutant shows attenuated seedling growth inhibition and cell features of programmed cell death upon FB1 exposure, indicating that MPK6 mediates signaling for FB1-triggered cell death [25].

In this study, we hypothesized that PTI suppresses PCD and investigated the effect of PTI on FB1-triggered cell death. We used FB1 as an inducer of PCD since FB1 simulates HR-like PCD and highly purified FB1 is commercially available. FB1-triggered cell death was suppressed by PTI triggered by flg22 or elf18 (flg22-, and elf18-PTI). FB1-triggered cell death and its suppression by flg22-PTI were observed in wild-type Col-0 and the defense related signaling mutants, *dde2*, *ein2*, *pad4*, *sid2* and *dde2*/*ein2*/*pad4*/*sid2* quadruple mutant. FB1triggered induction of *MKK4* and *MKK5* mRNA expression and phosphorylation of MPK3 and MPK6 at a late time point were inhibited by flg22 treatment. The suppression effect of flg22-PTI on FB1-triggered cell death was attenuated in *mpk6* T-DNA insertion mutants. These results suggest that suppression of the MAPK cascade by PTI signaling results in suppression of FB1-triggered cell death and that prolonged activation of the MAPKs leads to eventually HR like cell death.

## Results and Discussion

### PTI suppresses FB1-triggered cell death

Flg22-PTI can strongly suppress the growth of a virulent *Pseudomonas syringae* strain if leaves are pretreated with flg22 one day prior to inoculation with bacteria, but no PCD is evident [9], [26]. Thus, strong immunity is not necessarily correlated with PCD, unlike an HR during ETI. We speculated that plants have a mechanism to avoid excessive PCD after sufficient activation of PTI. To investigate this hypothesis, we used FB1 as PCD inducer for the following reasons. (1) PCD triggered by bacterial strains expressing effectors that are recognized by R proteins was unsuitable to test the direct effect of PTI on PCD because delivery of effectors by the bacterial type III secretion system is suppressed by PTI [18]. (2) *In planta* expression of such ETI-triggering effectors using an established chemical inducible expression system, such as dexamethasone [27] and estradiol [28] inducible systems, was inhibited by PTI (Figure S1A, B). This inhibitory effect of PTI was not transgene-specific since induced expression of an arbitrary reporter, the β-glucuronidase (GUS) gene, was also inhibited. (Figure S1C). Therefore, induced expression of ETI-triggering effector genes was not suitable for the analysis of the PTI and PCD interaction. (3) FB1 develops lesions reminiscent of the HR [21], [29].

**Figure 1:**
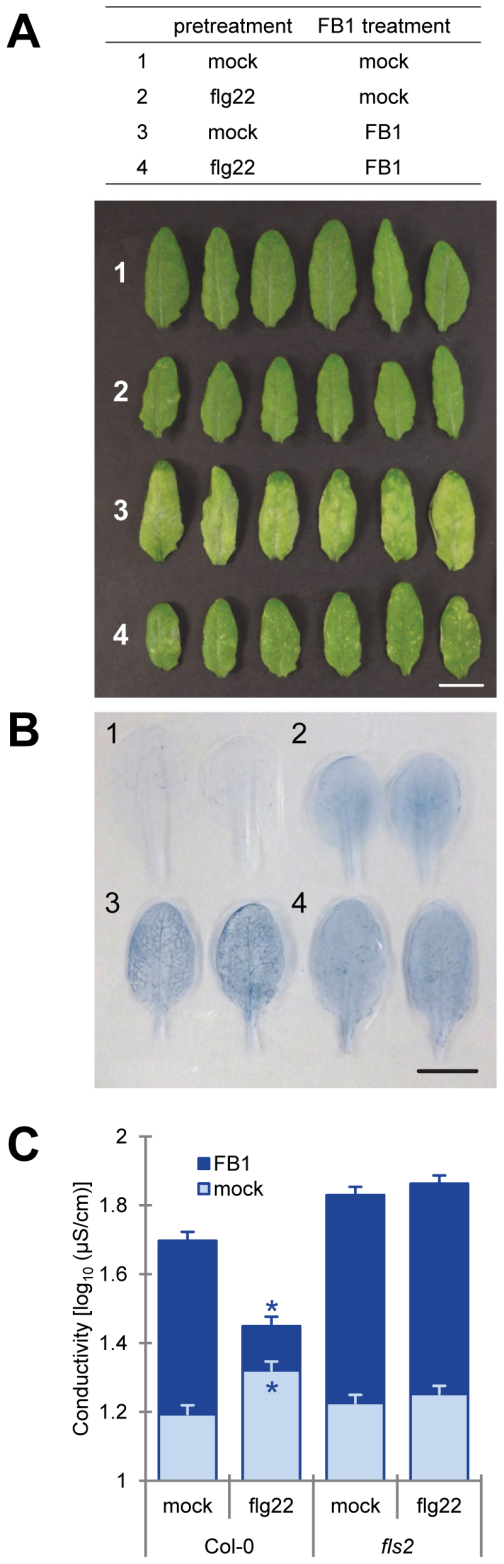
Flg22 pretreatment suppressed FB1-triggered cell death. Twenty-four hours after pretreatment with water (mock) or 1 µM flg22, 1% methanol (mock) or 50 µM FB1 were infiltrated into the pretreated leaves of four-week old plants. Three days after FB1 treatment, the leaves were subjected to macroscopic examination (A) or trypan blue staining (B) to visualizeFB1-triggered cell death. Scale bar  =  1 cm. (C) Quantification of FB1-triggered cell death by electrolyte leakage. Leaf disks (6 mm diameter) were prepared 48 hours after FB1 treatment. Four leaf disks, from two leaves, were floated on in wells of 12-well plate each containing 2 ml of purified water. Bars represent means and standard errorsfor biological replicates, calculated using a mixed linear model: four to six biological replicates were performed for each of two independent experiments. The vertical axis shows the log_10_-transformed conductivity values. Asterisks indicate significant differences from the mock-pretreatment (*P*<0.001, two-tailed *t*-tests).

FB1-triggered cell death was macroscopically observed as yellowing of leaves three to four days after inoculation of Arabidopsis mature leaves with 50 µM FB1 (Figure 1A). Leaves pretreated with 1 µM flg22 24 hours prior to FB1 treatment showed a reduction of FB1-triggered cell death visible as a lower level of leaf yellowing. This attenuation of PCD was confirmed when we analyzed the extent of cell death using trypan blue staining (Figure 1B) and electrolyte leakage measurements (Figure 1C). Flg22 pretreatment did not suppress FB1-triggered cell death in *fls2*, a flg22-receptor mutant, indicating that the flg22 effect depends on its specific receptor (Figure 1C).

**Figure 2:**
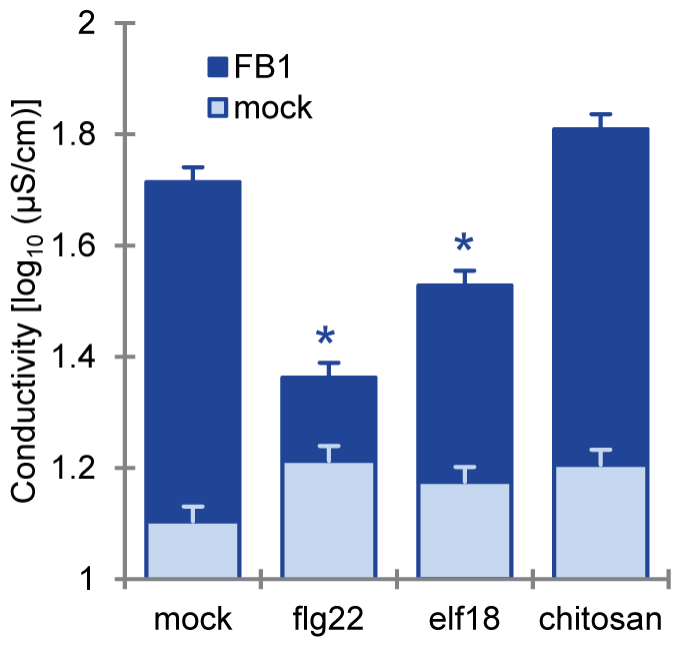
Chitosan treatment did not suppress FB1-triggered cell death. FB1-triggered cell death was quantified by electrolyte leakage measurements. This experiment was performed as described in Figure 1C except for the following differences. Twenty-four hours after pretreatment with water (mock), 1 µM flg22, 1 µM elf18 or 100 µg/ml chitosan, 1% methanol (mock) or 50 µM FB1 or were infiltrated into the pretreated leaves of four-week old plants. Bars represent means and standard errors for biological replicates, calculated using a mixed linear model: three to six biological replicates wereperformed for each of two independent experiments.

We also tested PCD suppression by two other MAMPs, elf18 and chitin. We used chitosan, a partially de-acetylated, readily water-soluble derivative of chitin, in place of chitin. Chitosan induces responses very similar to those induced by chitin, and the response is dependent on CERK1, the chitin receptor, in Arabidopsis (Figure S2) [30]. To ensure that chitosan treatment induced PTI, we examined the expression of PTI inducible genes. As shown in Figure S2, 1 µM flg22, 1 µM elf18, or 100 µg/ml chitosan treatment similarly induced the mRNA levels of *Chitinase* (At2g43620) and *PR6* (At2g38870). Despite the similar induction of the marker genes, chitosan pretreatment did not suppress FB1-triggered cell death, whereas elf18 pretreatment did (Figure 2). FB1 is a mycotoxin produced by several species of Fusarium molds which have cell walls containing chitin and other polysaccharides [31]. The observation that FB1-triggered cell death is not affected by chitin-PTI suggests that the fungal toxin evolved to induce a PCD that is not inhibited by chitin-PTI. Otherwise FB1 would not be able to induce a PCD as fungi are coated with chitin containing cell walls.

Since flg22-PTI is well characterized, we focused on the flg22 effect for further analysis. Suppression of FB1-triggered cell death by flg22 was observed when flg22 was administered 48 or 0 hours prior to FB1 treatment (Figure S3A). To investigate whether the flg22 effect is dose-dependent, we co-infiltrated leaves with various concentrations of flg22 and FB1. As shown in Figure S3B, treatment with 50 nM flg22 was sufficient to suppress FB1-triggered cell death. The fact that PCD suppression can be induced with very low amounts of flg22 suggests that the suppression is a biologically relevant phenomenon.

### FB1-triggered cell death in hormone signaling mutants

**Figure 3:**
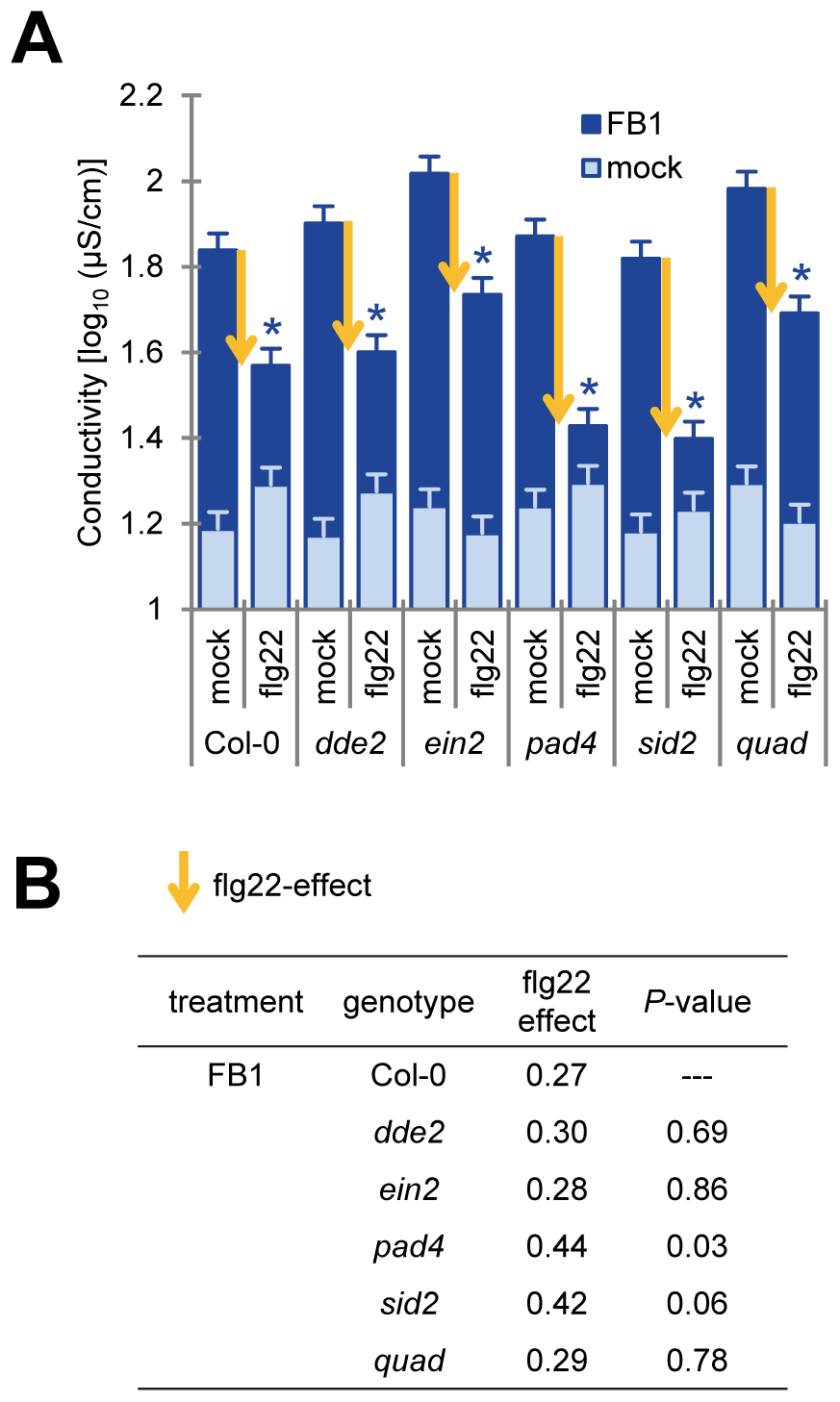
Flg22 pretreatment suppressed FB1-triggered cell death in *dde2*, *ein2*, *pad4*, *sid2* and quadruple mutants. (A) Quantification of FB1-triggered cell death by electrolyte leakage. This experiment was performed as described in Figure 1C except for the following differences. Twenty-four hours after pretreatment with water (mock) or 1 µM flg22, 1% methanol (mock) or 50 µM FB1 was infiltrated into the pretreated leaves of four-week old plants. Bars represent means and standard errors for biological replicates, calculated using a mixed linear model: four biological replicates were performed foreach of two independent experiments. Yellow arrows indicate the strength of the flg22 effect. Asterisks indicate significant differences from mock (*P*<0.001, two-tailed *t*-tests). (B) Comparison of the flg22 effect in the mutants for FB1-triggered cell death. The flg22 effect was estimated by subtracting the electrolyte leakage value of the flg22-treated sample from the value of the corresponding mock-treated sample. The flg22 effect in each mutant was compared with that in Col-0 using a two-tailed *t*-test using the standard errors calculated based on the mixed linear model to obtain the *P*-values. Quadruple mutant of *dde2/ein2/pad4/sid2* was shown as *quad*.

The plant signaling molecules jasmonic acid (JA), ethylene (ET) and salicylic acid (SA) play important roles in plant immune responses [32], [33]. Interactions between the SA and the JA/ET signaling sectors can be both antagonistic and synergistic [9], [34], [35], [36]. These three signaling sectors are involved in PCD triggered by FB1 or oxidative stress [29], [34], [37]. To investigate the involvement of these sectors in PTI suppression of FB1-triggered cell death, we measured electrolyte leakage after FB1 treatment in *dde2*, *ein2*, *pad4*, and *sid2* single mutants as well as in a *dde2/ein2/pad4/sid2* quadruple mutant. DDE2, EIN2 and SID2 are essential components of the JA, ET and SA signaling sectors, respectively. The *pad4* mutation affects the SA sector and expression of many SA-independent pathogen-induced genes [9], [14], [38]. As shown in Figure 3A, FB1-triggered cell death estimated by electrolyte leakage was observed after FB1 treatment of each single and the quadruple mutant in mature leaves. A previous report indicated that FB1-triggered cell death requires JA, ET and SA-dependent signals in protoplasts [29]. This suggests that FB1-triggered induction of cell death is not the same between intact leaves and protoplasts. Additionally, suppression of FB1-triggered cell death by flg22 was observed in each single and the quadruple mutant. The flg22 effect, which was estimated by subtracting the value of electrolyte leakage of flg22-pretreated leaves from the value of mock-pretreated leaves, was slightly different between Col-0 and *pad4* (Figure 3B, *P* = 0.03), suggesting that PAD4 might play a minor role in mediating the flg22 effect. It has been reported that ethylene signaling controls *FLS2* gene expression and responses to flg22 are impaired in *ein2* [39]. However, our previous work showed that early signaling events in response to flg22, such as MAPK activation and induction of MAMP-responsive gene expression, were intact [9] in the quadruple mutant. Taken together, the work cited above and our results that treatment with 50 nM flg22 suppressed FB1-triggered cell death (Figure S3b), indicate that early flg22-responses, which are JA, ET and SA independent, may be important for suppression of FB1-triggered cell death in intact adult leaves.

### FB1 induced activation of MAPKs was affected by PTI

**Figure 4:**
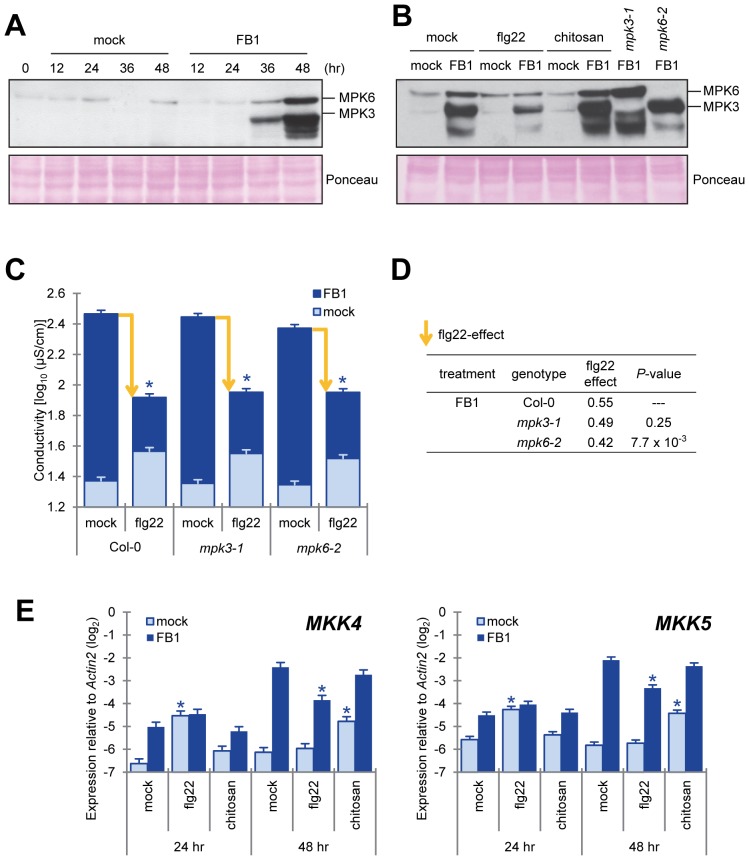
Flg22 treatment inhibited the MKK4/MKK5-MPK6 pathway, which positively regulated FB1-triggered cell death. (A, B) Activation of MAPKs by FB1 treatment. The fifth and sixth true leaf of three-week old plants were treated with water (mock), 1 µM flg22 or 100 µg/ml chitosan and 1% methanol (mock) or 50 µM FB1, and samples were collected at 0 to 48 (A) or 48 (B) hours after treatment as indicated. Each sample was a pool of four to six leaves. Activated MAP kinases were detected by immunoblotting using the anti-phospho-p44/42 MAPK antibody. Equal loading of samples was confirmed by Ponceau S staining. Experiments were conducted twice with similar results. (C) Quantification of FB1-triggered cell death by electrolyte leakage. The sixth true leaf of three-week old plants was treated with water (mock) or 1 µM flg22 and 1% methanol (mock) or 50 µM FB1. Leaf disks (7 mm diameter) were prepared 48 hours after FB1 treatment. One leaf disk, from one leaf was floated in wells of 48-well plates each containing 0.5 ml of purified water. Bars represent means and standard errors for biological replicates, calculated using a mixed linear model: eight biological replicates were performed for each of two independent experiments. The vertical axis shows log_10_-transformed conductivity values. Yellow arrows indicate the levels of the flg22 effect. Asterisks indicate significant differences from mock (*P*<0.001, two-tailed *t*-tests). (D) Comparison of the flg22 effect in MAPK mutants for the FB1-triggered cell death. The flg22 effect was estimated by subtracting the electrolyte leakage values of flg22-treated samples from the values of mock-treated samples. The flg22 effect on each mutant was compared with that on Col-0 using a two-tailed *t*-test using the standard errors calculated based on the mixed linear model to obtain the *P*-values. (E) Induction of *MKK4* and *MKK5* mRNA expression by FB1. The fifth and sixth true leaves of three-week old plants were treated with water (mock), 1 µM flg22 or 100 µg/ml chitosan (chitin) and 1% methanol or 50 µM FB1, and samples were collected at 24 and 48 hours after treatment. Each sample was a pool of four leaves from two plants. The mRNA levels of *MKK4* and *MKK5* were measured by qRT-PCR. Bars represent means and standard errors for biological replicates, calculated using a mixed linear model: two or three biological replicates were performed foreach of two independent experiments. The vertical axis is the log_2_ expression level relative to that of *Actin2* (At3g18780). Asterisks indicate significant differences from mock treatment (*P*<0.001, two-tailed *t*-tests).

FB1 activates MAPK cascades in plant and animal cells [25], [40], [41]. Growth suppression of seedlings on an FB1-containing medium was reduced in a T-DNA insertion mutant of *MPK6* [25]. Perception of flg22 or AvrRpt2, an ETI-triggering effector, by the cognate receptor triggers phosphorylation of MPK3 and MPK6 in a transient or sustained manner, respectively [9], [42]. Since FB1-triggered cell death is macroscopically observed 3–4 days after FB1 treatment, we hypothesized that MPK3 and MPK6 are activated by FB1 at late time points in a sustained manner. As shown in Figure 4A, phosphorylation of MPK3, MPK6 and other MPKs were detected 36–48 hours after FB1 treatment. Interestingly, flg22 treatment inhibited FB1-triggered MPK3 and MPK6 phosphorylation but chitosan treatment did not (Figure 4B). To determine whether MPK3 and MPK6 signaling are essential for FB1-triggered cell death and its suppression by flg22, we measured electrolyte leakage after co-infiltration of FB1 and flg22 in *mpk3-1* and *mpk6-2* T-DNA insertion mutants.

In *mpk6-2*, but not in *mpk3-1*, FB1-triggered cell death was attenuated (*P* = 0.0055) (Figure 4C). This result is consistent with previous reports showing MPK6 plays a role in cell death triggered by FB1 and heat shock [25], [43]. The flg22 effect in *mpk6-2*, which was estimated by subtracting the ion conductivity value for FB1 with flg22 treatment from that for FB1 only treatment, was significantly smaller (23%) than that in Col-0 (Figure 4D). This was also observed with another *mpk6* mutant allele (*mpk6-3*, Figure S4). Since both FB1-triggered cell death and the flg22 effect were not completely abolished in *mpk6* mutants, we concluded that MPK6-dependent as well as MPK6-independent signaling pathways are involved in these responses. MPK3 may be involved, as MPK3 and MPK6 function redundantly in some biological processes [23], [44].

In the Arabidopsis genome, there are 10 predicted MAPKKs [45]. Previous studies showed that MKK4 and MKK5 are the upstream kinases activating MPK3 and MPK6 during abiotic and biotic stress-response signaling [23], [45], [46]. Our previous mRNA profiling data showed that mRNA levels of *MKK4* increased during infection with an ETI-triggering bacterial strain, *Pto* DC3000 AvrRpt2 [47]. The *MKK4* and *MKK5* mRNA levels increased 24 hours after flg22 treatment and 48 hours after FB1 treatment (Figure 4E). The induced *MKK4* and *MKK5* mRNA levels were higher with FB1 treatment than with flg22 treatment. Flg22 treatment reduced the FB1-induced levels of *MKK4* and *MKK5* mRNA, whereas chitosan treatment did not (Figure 4E). Induced expression of constitutively active forms of MKK4 and MKK5 leads to HR-like cell death [24]. Therefore, late and prolonged *MKK4* and *MKK5* mRNA induction by FB1 treatment may be important for FB1-triggered cell death, and reduction of FB1-induced *MKK4* and *MKK5* mRNA levels by flg22 treatment may be a cause of attenuation of FB1-triggered cell death by flg22 treatment.

Vacuolar processing enzymes (VPEs) play an important role for both FB1-triggered cell death and heat shock triggered PCD (HS-PCD) [22], [43]. *VPE* mRNAs increase after FB1 and HS treatment [22], [43]. Heat shock activates the MAPK cascade, and MPK6 is important for heat shock induction of the *VPE* genes [43]. We found that flg22 treatment did not reduce the FB1-induced level of *γVPE* gene expression (Figure S5A). In contrast with HS treatment, FB1 induction of *γVPE* gene expression was not suppressed by a knockout mutation of MPK6 (Figure S5B). These results indicate that FB1- and HS-PCD use different signaling mechanisms for cell death induction, and that induced expression of *VPEs* is not sufficient to induce cell death.

**Figure 5:**
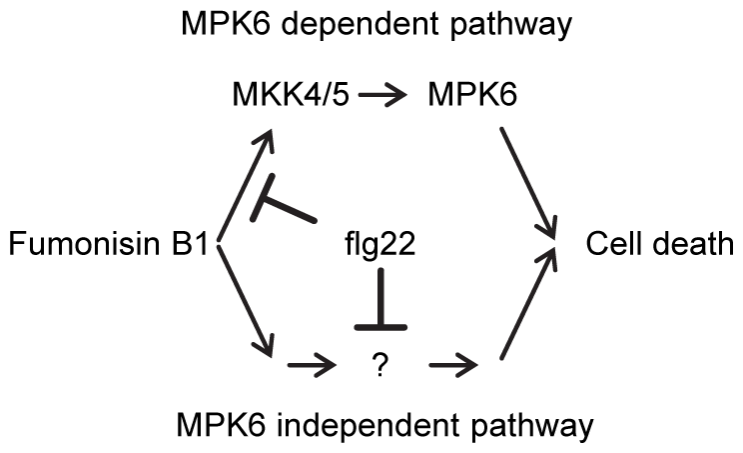
A proposed model for the FB1-triggered cell death and suppression by flg22- PTI. The MKK4/MKK5-MPK6 pathway partially contributes to FB1-triggered cell death. PTI may suppress FB1-triggered cell death by suppression of *MKK4* gene expression and by other so far unknown pathways.

In conclusion, our observations suggest that suppression of FB1-triggered cell death by flg22 treatment is partially mediated by flg22-triggered interference with late and prolonged induction of *MKK4* and *MKK5* gene expression (Figure 5), which affects signaling mediated by the downstream MAPK MPK6.

We hypothesize that suppression of PCD by PTI is not exclusive to FB1-triggered cell death since this system seems to benefit plants by avoiding excessive and wasteful PCD responses. It will be interesting to investigate the effect of PTI on ETI-associated PCD (i.e., the HR) and, if an effect is detectable, the factors that are involved in mediating the PTI effect. Comparison of such results with what we learned about the PTI effect on FB1-triggered cell death will deepen our understanding of signaling mechanisms involved in plant PCD.

## Materials and Methods

### Plant materials and growth conditions

*Arabidopsis thaliana* accession Col-0 was the background of all mutants used in this study. Arabidopsis mutants, *dde2-2* [10], *ein2-1* [11], *fls2* (SAIL_691C4) [7], *mpk3-1* (SALK151594) [48], *mpk6-2* (SALK_073907) [49], *mpk6-3* (SALK127507) [48], *pad4-1* [13], *sid2-2* [12] and d*de2-2*/*ein2-1*/*pad4-1*/*sid2-2* [9] were previously described.

Plants were grown in an environment-controlled chamber at 22°C with 75% relative humidity and a 12h/12h light/dark cycle.

### Chemicals

Flg22 and elf18 peptides were purchased from EZBiolab Inc. (Westfield, IN, USA).

Polymeric chitosan was used as inducer of chitin-PTI. Chitosan (C3646; SIGMA, St. Louis, MO, USA) was dissolved in 0.05 % (v/v) acetate to make 2 mg/ml solution and diluted in water for inoculation (final concentration: 100 µg/ml). Fumonisin B1 (F1147; SIGMA) was dissolved in methanol to make 5 mM FB1 and diluted in water for inoculation (final concentration: 50 µM). One percent methanol (v/v) was used as a negative control for FB1 treatment.

### Trypan blue staining

Trypan blue staining stock solution was 0.1% (w/v) trypan blue in solvent consisting of equal volumes of water saturated-phenol, lactate, glycerol and water. One volume of this stock solution was diluted with 2 volumes of 95% ethanol before use. Leaves were boiled in the diluted trypan blue staining solution for 1 minute and destained in chloral hydrate solution (1.25 g/ml chloral hydrate in water) for 2 days.

### Electrolyte leakage analysis

Three- or four-week old plants were infiltrated with 50 µM FB1. Forty-eight hours after infiltration, leaf disks (6 or 7 mm diameter) were made and floated in purified water for 48 hours under 12h/12h light/dark conditions. Electrolyte leakage values were determined by measuring conductivity with an electrical conductivity meter (B-173, Horiba, Japan).

Log_10_-transformed conductivity values were analyzed. The following mixed-effect model was fit to the log_10_-transformed conductivity data using the lme function of the nlme package in the R environment (http://www.r-project.org): *C_gptr_*  =  *GPT_gpt_*+*R_r_*+*ε_gptr_*, where *C*, log_10_-transformed conductivity; *GPT*, genotype:pretreatment:treatment interaction and random factors; *R*, independent experiment, *ε*, residual. The mean estimate of the genotype:pretreatment:treatment interaction was used as the modeled log_10_- transformed conductivity. The flg22-triggered suppression of electrolyte leakage was calculated by subtracting the electrolyte leakage values of the flg22-treated samples from the values of the mock-treated samples. Significance of differences was determined using two-tailed *t*-tests. For the *t*-tests, the standard error appropriate for each comparison was calculated using the variance and covariance values obtained from the model fitting. The raw data from the electrolyte leakage analysis are included in Table S2.

### Quantitative RT-PCR analysis

Total RNA was isolated using TRIzol (Invitrogen, Carlsbad, CA, USA) according to the manufacturer's protocol. Quantitative RT-PCR was performed using a Superscript III Platinum SYBR Green One-Step qRT-PCR kit (Invitrogen) and a LightCycler480 machine (Roche, Basel, Switzerland). Primers for qRT-PCR are listed in Table S1. The following mixed-effect model was fit to the Ct value data using the lme function of the nlme package in the R environment (http://www.r-project.org): *Ct_gtr_  =  GT_gt_+R_r_+ε_gtr_*, where *GT*, gene:treatment interaction and random factors; *R*, independent experiment; *ε*, residual. The mean estimates of the gene:treatment interaction were used as the modeled Ct values. The relative log_2_ expression values were obtained by subtracting the Ct value of the genes from the Ct value of the *Actin2* (At3g18780) gene and compared for each gene using two-tailed *t*-tests. For the *t*-tests, the standard error appropriate for each comparison was calculated using the variance and covariance values obtained from the model fitting.

### Measurement of MAPK activation

The activated MAPKs were detected as described previously [50].

The fifth and sixth leaves of three-week-old plants were infiltrated with 50 µM FB1. Samples, each of which was a pool of six leaves from three plants, were collected at each time point and flash frozen in liquid nitrogen. The frozen samples were ground in liquid nitrogen and homogenized in 100 µl of extraction buffer (100 mM HEPES, pH 7.5, 5 mM EDTA, 5 mM EGTA, 2 mM dithiothreitol, 10 mM Na_3_VO_4_, 10 mM NaF, 50 mM ß-glycerolphosphate, 1 mM phenylmethylsulfonyl fluoride, 1 tablet/10 ml extraction buffer of proteinase inhibitor cocktail (11 836 153 001; Roche Applied Science, Indianapolis, IN, USA) and phosphatase inhibitor cocktail (04 906 845 001; Roche Applied Science), 10% glycerol, 1% (w/v) polyvinylpolypyrrolidone). The protein concentration was determined using a Bradford assay (Bio-Rad, Hercules, CA, USA) with BSA as a standard. Sixty micrograms of protein per lane was separated in a 10% polyacrylamide gel. Immunoblot analysis was performed using anti-phospho-p44/42 MAPK (1∶2000, Cell Signaling Technology, Danvers, MA, USA) as the primary antibody, peroxidase-conjugated goat anti-rabbit IgG (1∶15,000, A6154; Sigma) as the secondary antibody, and Pierce Protein-Free T20 (TBS) Blocking Buffer (37351; Thermo Scientific, Waltham, MA, USA).

## Supporting Information

Figure S1 **Attenuation of chemical inducible gene expression system in plant.** Transgenic plants carrying the transgenes for estradiol (Ed)-inducible AvrRpt2 (parent vector: pER8), dexamethazone (Dex)-inducible AvrRpm1 (parent vector: pTA7002, gift from Jeff Dangl’s Lab) or estradiol (Ed)-inducible β-glucuronidase (GUS, parent vector: pER8) were used for this experiment. Twenty-four hours after pretreatment with water (mock) or 1 µM flg22, 10 µM estradiol (A, C) or dexamethazone (B) was infiltrated into the pretreated leaves of four-week old plants. The mRNA levels of *AvrRpt2* (A), *AvrRpm1* (B) or *GUS* (C) were measured by qRT-PCR. Bars represent means and standard errors for four (A) or three (B, C) biological replicates, calculated using a mixed linear model. The vertical axis is the log_2_-transformed expression level relative to that of *Actin2* (At3g18780). The level of expression in flg22-pretreated leaves was compared with that in mock-pretreated leaves using a two-tailed *t*-test using the standard errors calculated based on the mixed linear model to obtain the *P*-values. Expression levels below the dection limit of the qPCR system are shown as n.d. (not determined).
(PDF)

Figure S2 **Induction of** ***Chitinase*** **and** ***PR6*** **mRNA by flg22, elf18 and chitosan.** Four-week old plants were treated with 1 µM flg22, 1 µM elf18 or 100 µg/ml chitosan, and samples were collected 3 hours after MAMP treatment. Each sample was a pool of six mature leaves. The mRNA levels of *Chitinase* (At2g43620) and *PR6* (At2g38870) were measured by qRT-PCR. The receptor deficient mutants (*fls2*: SAIL_691C4, *efr-2*: SALK_068675, and *cerk1-2*: GABI_096F09) were included as a negative control for each MAMP. Bars and error bars represent means and standard deviation for three independent experiments. The vertical axis is the log_2_ expression level relative to that of *Actin2* (At3g18780).
(PDF)

Figure S3 **During and concentrations of flg22 treatment required for suppression of FB1-triggered cell death.** (A, B) Quantification of FB1-triggered cell death by electrolyte leakage. This experiment was performed as described in Figure 1C except for the following differences. Forty-eight hours (A) or 0 hour (A, B) after pretreatment with water (mock) or 50 nM (B) to 1 µM flg22 (A, B), 50 µM FB1was infiltrated into the pretreated leaves of four-week old plants. Bars represent means and standard errors for biological replicates, calculated using a mixed linear model: four to six biological replicates were performed for each of two independent experiments.
(PDF)

Figure S4 **Quantification of FB1-triggered cell death by electrolyte leakage in** ***mpk6-3*** **.** This experiment was performed as described in Figures 4C and D. Bars represent means and standard errors for biological replicates, calculated using a mixed linear model: five to six biological replicates were performed for each of two independent experiments.
(PDF)

Figure S5 **Induction of** ***γVPE*** **mRNA by FB1.** The fifth and sixth true leaves of three-week old plants were treated with water (mock), 1 µM flg22 or 100 µg/ml chitosan (chitin) and 1% methanol (mock) or 50 µM FB1, and samples were collected at 24 and 48 hours after treatment. Each sample was a pool of four leaves, from two plants each. The mRNA levels of *γVPE* were measured by qRT-PCR. Bars represent means and standard errors for biological replicates, calculated using a mixed linear model: two biological replicates were performed for each of two independent experiments. The vertical axis depicts the log_2_ transformed expression values relative to that of *Actin2* (At3g18780). Asterisks indicate significant differences between mock and MAMPs (A) or Col-0 and *mpk* mutants (B) (*P*<0.001, two-tailed *t*-tests).
(PDF)

Table S1 **The sequences of the primers used in quantitative RT-PCR.**
(XLSX)

Table S2 **The raw ion conductivity data used in the figures.**
(XLSX)

## References

[1] Beers EP, McDowell JM (2001) Regulation and execution of programmed cell death in response to pathogens, stress and developmental cues. Curr Opin Plant Biol 4: 561–567. 11641074 10.1016/s1369-5266(00)00216-8

[2] Boller T, Felix G (2009) A renaissance of elicitors: perception of microbe-associated molecular patterns and danger signals by pattern-recognition receptors. Annu Rev Plant Biol 60: 379–406. 19400727 10.1146/annurev.arplant.57.032905.105346

[3] Felix G, Duran JD, Volko S, Boller T (1999) Plants have a sensitive perception system for the most conserved domain of bacterial flagellin. Plant J 18: 265–276. 10377992 10.1046/j.1365-313x.1999.00265.x

[4] Kunze G, Zipfel C, Robatzek S, Niehaus K, Boller T, et al (2004) The N terminus of bacterial elongation factor Tu elicits innate immunity in Arabidopsis plants. Plant Cell 16: 3496–3507. 15548740 10.1105/tpc.104.026765 PMC535888

[5] Miya A, Albert P, Shinya T, Desaki Y, Ichimura K, et al (2007) CERK1, a LysM receptor kinase, is essential for chitin elicitor signaling in Arabidopsis. Proc Natl Acad Sci U S A 104: 19613–19618. 18042724 10.1073/pnas.0705147104 PMC2148337

[6] Wan J, Zhang XC, Neece D, Ramonell KM, Clough S, et al (2008) A LysM receptor-like kinase plays a critical role in chitin signaling and fungal resistance in Arabidopsis. Plant Cell 20: 471–481. 18263776 10.1105/tpc.107.056754 PMC2276435

[7] Zipfel C, Robatzek S, Navarro L, Oakeley EJ, Jones JD, et al (2004) Bacterial disease resistance in Arabidopsis through flagellin perception. Nature 428: 764–767. 15085136 10.1038/nature02485

[8] Zipfel C, Kunze G, Chinchilla D, Caniard A, Jones JD, et al (2006) Perception of the bacterial PAMP EF-Tu by the receptor EFR restricts Agrobacterium-mediated transformation. Cell 125: 749–760. 16713565 10.1016/j.cell.2006.03.037

[9] Tsuda K, Sato M, Stoddard T, Glazebrook J, Katagiri F (2009) Network properties of robust immunity in plants. PLoS Genet 5: e1000772. 20011122 10.1371/journal.pgen.1000772 PMC2782137

[10] von Malek B, van der Graaff E, Schneitz K, Keller B (2002) The Arabidopsis male-sterile mutant dde2-2 is defective in the ALLENE OXIDE SYNTHASE gene encoding one of the key enzymes of the jasmonic acid biosynthesis pathway. Planta 216: 187–192. 12430030 10.1007/s00425-002-0906-2

[11] Alonso JM, Hirayama T, Roman G, Nourizadeh S, Ecker JR (1999) EIN2, a bifunctional transducer of ethylene and stress responses in Arabidopsis. Science 284: 2148–2152. 10381874 10.1126/science.284.5423.2148

[12] Wildermuth MC, Dewdney J, Wu G, Ausubel FM (2001) Isochorismate synthase is required to synthesize salicylic acid for plant defence. Nature 414: 562–565. 11734859 10.1038/35107108

[13] Jirage D, Tootle TL, Reuber TL, Frost LN, Feys BJ, et al (1999) Arabidopsis thaliana PAD4 encodes a lipase-like gene that is important for salicylic acid signaling. Proc Natl Acad Sci U S A 96: 13583–13588. 10557364 10.1073/pnas.96.23.13583 PMC23991

[14] Glazebrook J, Chen W, Estes B, Chang HS, Nawrath C, et al (2003) Topology of the network integrating salicylate and jasmonate signal transduction derived from global expression phenotyping. Plant J 34: 217–228. 12694596 10.1046/j.1365-313x.2003.01717.x

[15] Newman MA, von Roepenack E, Daniels M, Dow M (2000) Lipopolysaccharides and plant responses to phytopathogenic bacteria. Mol Plant Pathol 1: 25–31. 20572947 10.1046/j.1364-3703.2000.00004.x

[16] Klement Z, Bozso Z, Kecskes ML, Besenyei E, Arnold C, et al (2003) Local early induced resistance of plants as the first line of defence against bacteria. Pest Manag Sci 59: 465–474. 12701709 10.1002/ps.694

[17] Oh HS, Collmer A (2005) Basal resistance against bacteria in Nicotiana benthamiana leaves is accompanied by reduced vascular staining and suppressed by multiple Pseudomonas syringae type III secretion system effector proteins. Plant J 44: 348–359. 16212612 10.1111/j.1365-313X.2005.02529.x

[18] Crabill E, Joe A, Block A, van Rooyen JM, Alfano JR (2010) Plant immunity directly or indirectly restricts the injection of type III effectors by the Pseudomonas syringae type III secretion system. Plant Physiol 154: 233–244. 20624999 10.1104/pp.110.159723 PMC2938138

[19] Tolleson WH, Melchior WB Jr, Morris SM, McGarrity LJ, Domon OE, et al (1996) Apoptotic and anti-proliferative effects of fumonisin B1 in human keratinocytes, fibroblasts, esophageal epithelial cells and hepatoma cells. Carcinogenesis 17: 239–249. 8625445 10.1093/carcin/17.2.239

[20] Wang W, Jones C, Ciacci-Zanella J, Holt T, Gilchrist DG, et al (1996) Fumonisins and Alternaria alternata lycopersici toxins: sphinganine analog mycotoxins induce apoptosis in monkey kidney cells. Proc Natl Acad Sci U S A 93: 3461–3465. 8622958 10.1073/pnas.93.8.3461 PMC39631

[21] Stone JM, Heard JE, Asai T, Ausubel FM (2000) Simulation of fungal-mediated cell death by fumonisin B1 and selection of fumonisin B1-resistant (fbr) Arabidopsis mutants. Plant Cell 12: 1811–1822. 11041878 10.1105/tpc.12.10.1811 PMC149121

[22] Kuroyanagi M, Yamada K, Hatsugai N, Kondo M, Nishimura M, et al (2005) Vacuolar processing enzyme is essential for mycotoxin-induced cell death in Arabidopsis thaliana. J Biol Chem 280: 32914–32920. 16043487 10.1074/jbc.M504476200

[23] Asai T, Tena G, Plotnikova J, Willmann MR, Chiu WL, et al (2002) MAP kinase signalling cascade in Arabidopsis innate immunity. Nature 415: 977–983. 11875555 10.1038/415977a

[24] Ren D, Yang H, Zhang S (2002) Cell death mediated by MAPK is associated with hydrogen peroxide production in Arabidopsis. J Biol Chem 277: 559–565. 11687590 10.1074/jbc.M109495200

[25] Saucedo-Garcia M, Guevara-Garcia A, Gonzalez-Solis A, Cruz-Garcia F, Vazquez-Santana S, et al (2011) MPK6, sphinganine and the LCB2a gene from serine palmitoyltransferase are required in the signaling pathway that mediates cell death induced by long chain bases in Arabidopsis. New Phytol 191: 943–957. 21534970 10.1111/j.1469-8137.2011.03727.x

[26] Katagiri F, Tsuda K (2010) Understanding the plant immune system. Mol Plant Microbe Interact 23: 1531–1536. 20653410 10.1094/MPMI-04-10-0099

[27] Aoyama T, Chua NH (1997) A glucocorticoid-mediated transcriptional induction system in transgenic plants. Plant J 11: 605–612. 9107046 10.1046/j.1365-313x.1997.11030605.x

[28] Zuo J, Niu QW, Chua NH (2000) Technical advance: An estrogen receptor-based transactivator XVE mediates highly inducible gene expression in transgenic plants. Plant J 24: 265–273. 11069700 10.1046/j.1365-313x.2000.00868.x

[29] Asai T, Stone JM, Heard JE, Kovtun Y, Yorgey P, et al (2000) Fumonisin B1-induced cell death in arabidopsis protoplasts requires jasmonate-, ethylene-, and salicylate-dependent signaling pathways. Plant Cell 12: 1823–1836. 11041879 10.1105/tpc.12.10.1823 PMC149122

[30] Petutschnig EK, Jones AM, Serazetdinova L, Lipka U, Lipka V (2010) The lysin motif receptor-like kinase (LysM-RLK) CERK1 is a major chitin-binding protein in Arabidopsis thaliana and subject to chitin-induced phosphorylation. J Biol Chem 285: 28902–28911. 20610395 10.1074/jbc.M110.116657 PMC2937917

[31] Desjardins AE, Plattner RD, Nelsen TC, Leslie JF (1995) Genetic analysis of fumonisin production and virulence of Gibberella fujikuroi mating population A (Fusarium moniliforme) on maize (Zea mays) seedlings. Appl Environ Microbiol 61: 79–86. 7887628 10.1128/aem.61.1.79-86.1995 PMC167262

[32] van Wees SC, de Swart EA, van Pelt JA, van Loon LC, Pieterse CM (2000) Enhancement of induced disease resistance by simultaneous activation of salicylate- and jasmonate-dependent defense pathways in Arabidopsis thaliana. Proc Natl Acad Sci U S A 97: 8711–8716. 10890883 10.1073/pnas.130425197 PMC27013

[33] Glazebrook J (2005) Contrasting mechanisms of defense against biotrophic and necrotrophic pathogens. Annu Rev Phytopathol 43: 205–227. 16078883 10.1146/annurev.phyto.43.040204.135923

[34] Mur LA, Kenton P, Atzorn R, Miersch O, Wasternack C (2006) The outcomes of concentration-specific interactions between salicylate and jasmonate signaling include synergy, antagonism, and oxidative stress leading to cell death. Plant Physiol 140: 249–262. 16377744 10.1104/pp.105.072348 PMC1326048

[35] Smith JL, De Moraes CM, Mescher MC (2009) Jasmonate- and salicylate-mediated plant defense responses to insect herbivores, pathogens and parasitic plants. Pest Manag Sci 65: 497–503. 19206090 10.1002/ps.1714

[36] Thaler JS, Humphrey PT, Whiteman NK, (2012) Evolution of jasmonate and salicylate signal crosstalk. Trends Plant Sci. 2012 Apr :10. 10.1016/j.tplants.2012.02.010 22498450

[37] Devadas SK, Enyedi A, Raina R (2002) The Arabidopsis hrl1 mutation reveals novel overlapping roles for salicylic acid, jasmonic acid and ethylene signalling in cell death and defence against pathogens. Plant J 30: 467–480. 12028576 10.1046/j.1365-313x.2002.01300.x

[38] Wang L, Mitra RM, Hasselmann KD, Sato M, Lenarz Wyatt L, et al (2008) The genetic network controlling the Arabidopsis transcriptional response to Pseudomonas syringae pv. maculicola: roles of major regulators and the phytotoxin coronatine. Mol Plant Microbe Interact 21: 1408–1420. 18842091 10.1094/MPMI-21-11-1408

[39] Boutrot F, Segonzac C, Chang KN, Qiao H, Ecker JR, et al (2010) Direct transcriptional control of the Arabidopsis immune receptor FLS2 by the ethylene-dependent transcription factors EIN3 and EIL1. Proc Natl Acad Sci U S A 107: 14502–14507. 20663954 10.1073/pnas.1003347107 PMC2922558

[40] Wattenberg EV, Badria FA, Shier WT (1996) Activation of mitogen-activated protein kinase by the carcinogenic mycotoxin fumonisin B1. Biochem Biophys Res Commun 227: 622–627. 8878562 10.1006/bbrc.1996.1555

[41] Pinelli E, Poux N, Garren L, Pipy B, Castegnaro M, et al (1999) Activation of mitogen-activated protein kinase by fumonisin B(1) stimulates cPLA(2) phosphorylation, the arachidonic acid cascade and cAMP production. Carcinogenesis 20: 1683–1688. 10469611 10.1093/carcin/20.9.1683

[42] Underwood W, Zhang S, He SY (2007) The Pseudomonas syringae type III effector tyrosine phosphatase HopAO1 suppresses innate immunity in Arabidopsis thaliana. Plant J 52: 658–672. 17877704 10.1111/j.1365-313X.2007.03262.x

[43] Li Z, Yue H, Xing D, (2012) MAP Kinase 6-mediated activation of vacuolar processing enzyme modulates heat shock-induced programmed cell death in Arabidopsis. New Phytol. 2012 Apr 12. doi:10.1111/j.1469-8137. 10.1111/j.1469-8137.2012.04131.x 22497243

[44] Ren D, Liu Y, Yang KY, Han L, Mao G, et al (2008) A fungal-responsive MAPK cascade regulates phytoalexin biosynthesis in Arabidopsis. Proc Natl Acad Sci U S A 105: 5638–5643. 18378893 10.1073/pnas.0711301105 PMC2291085

[45] Nakagami H, Pitzschke A, Hirt H (2005) Emerging MAP kinase pathways in plant stress signalling. Trends Plant Sci 10: 339–346. 15953753 10.1016/j.tplants.2005.05.009

[46] Pedley KF, Martin GB (2005) Role of mitogen-activated protein kinases in plant immunity. Curr Opin Plant Biol 8: 541–547. 16043387 10.1016/j.pbi.2005.07.006

[47] Sato M, Tsuda K, Wang L, Coller J, Watanabe Y, et al (2010) Network modeling reveals prevalent negative regulatory relationships between signaling sectors in Arabidopsis immune signaling. PLoS Pathog 6: e1001011. 20661428 10.1371/journal.ppat.1001011 PMC2908620

[48] Bethke G, Pecher P, Eschen-Lippold L, Tsuda K, Katagiri F, et al (2012) Activation of the Arabidopsis thaliana mitogen-activated protein kinase MPK11 by the flagellin-derived elicitor peptide, flg22. Mol Plant Microbe Interact 25: 471–480. 22204645 10.1094/MPMI-11-11-0281

[49] Liu Y, Zhang S (2004) Phosphorylation of 1-aminocyclopropane-1-carboxylic acid synthase by MPK6, a stress-responsive mitogen-activated protein kinase, induces ethylene biosynthesis in Arabidopsis. Plant Cell 16: 3386–3399. 15539472 10.1105/tpc.104.026609 PMC535880

[50] Ahlfors R, Macioszek V, Rudd J, Brosche M, Schlichting R, et al (2004) Stress hormone-independent activation and nuclear translocation of mitogen-activated protein kinases in Arabidopsis thaliana during ozone exposure. Plant J 40: 512–522. 15500467 10.1111/j.1365-313X.2004.02229.x

