# Supplementary figures and images for: Pattern-Triggered Immunity Suppresses Programmed Cell Death Triggered by Fumonisin B1
Source: PLoS One. 2013 Apr 1;8(4):e60769. doi: 10.1371/journal.pone.0060769 (PMC3613394; doi:10.1371/journal.pone.0060769)

## Supporting Information Figure S1

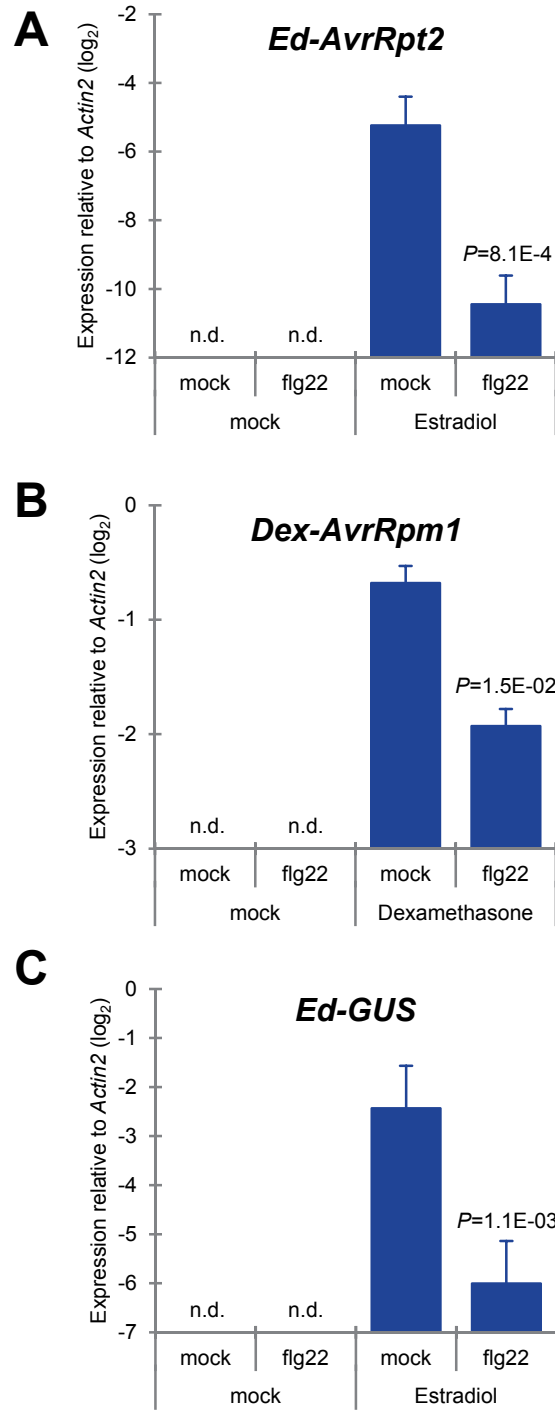

Supplement: Figure S1 — Attenuation of chemical inducible gene expression system in plant. Transgenic plants carrying the transgenes for estradiol (Ed)-inducible AvrRpt2 (parent vector: pER8), dexamethazone (Dex)-inducible AvrRpm1 (parent vector: pTA7002, gift from Jeff Dangl’s Lab) or estradiol (Ed)-inducible β-glucuronidase (GUS, parent vector: pER8) were used for this experiment. Twenty-four hours after pretreatment with water (mock) or 1 µM flg22, 10 µM estradiol (A, C) or dexamethazone (B) was infiltrated into the pretreated leaves of four-week old plants. The mRNA levels of AvrRpt2 (A), AvrRpm1 (B) or GUS (C) were measured by qRT-PCR. Bars represent means and standard errors for four (A) or three (B, C) biological replicates, calculated using a mixed linear model. The vertical axis is the log2-transformed expression level relative to that of Actin2 (At3g18780). The level of expression in flg22-pretreated leaves was compared with that in mock-pretreated leaves using a two-tailed t-test using the standard errors calculated based on the mixed linear model to obtain the P-values. Expression levels below the dection limit of the qPCR system are shown as n.d. (not determined). (PDF) [file pone.0060769.s001.pdf]

## Supporting Information Figure S2

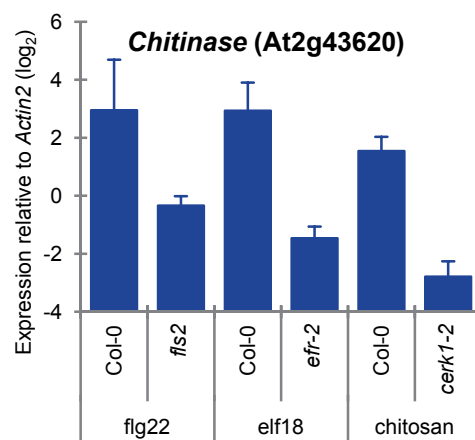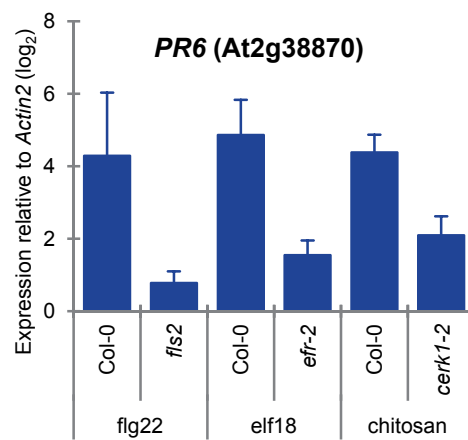

Supplement: Figure S2 — Induction of Chitinase and PR6 mRNA by flg22, elf18 and chitosan. Four-week old plants were treated with 1 µM flg22, 1 µM elf18 or 100 µg/ml chitosan, and samples were collected 3 hours after MAMP treatment. Each sample was a pool of six mature leaves. The mRNA levels of Chitinase (At2g43620) and PR6 (At2g38870) were measured by qRT-PCR. The receptor deficient mutants (fls2: SAIL_691C4, efr-2: SALK_068675, and cerk1-2: GABI_096F09) were included as a negative control for each MAMP. Bars and error bars represent means and standard deviation for three independent experiments. The vertical axis is the log2 expression level relative to that of Actin2 (At3g18780). (PDF) [file pone.0060769.s002.pdf]

## Supporting Information Figure S3

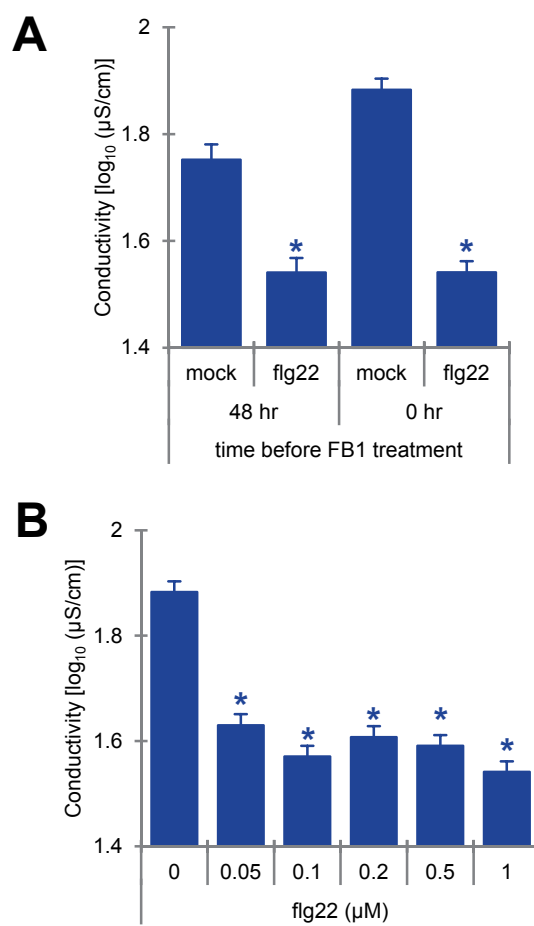

Supplement: Figure S3 — During and concentrations of flg22 treatment required for suppression of FB1-triggered cell death. (A, B) Quantification of FB1-triggered cell death by electrolyte leakage. This experiment was performed as described in Figure 1C except for the following differences. Forty-eight hours (A) or 0 hour (A, B) after pretreatment with water (mock) or 50 nM (B) to 1 µM flg22 (A, B), 50 µM FB1was infiltrated into the pretreated leaves of four-week old plants. Bars represent means and standard errors for biological replicates, calculated using a mixed linear model: four to six biological replicates were performed for each of two independent experiments. (PDF) [file pone.0060769.s003.pdf]

## Supporting Information Figure S5

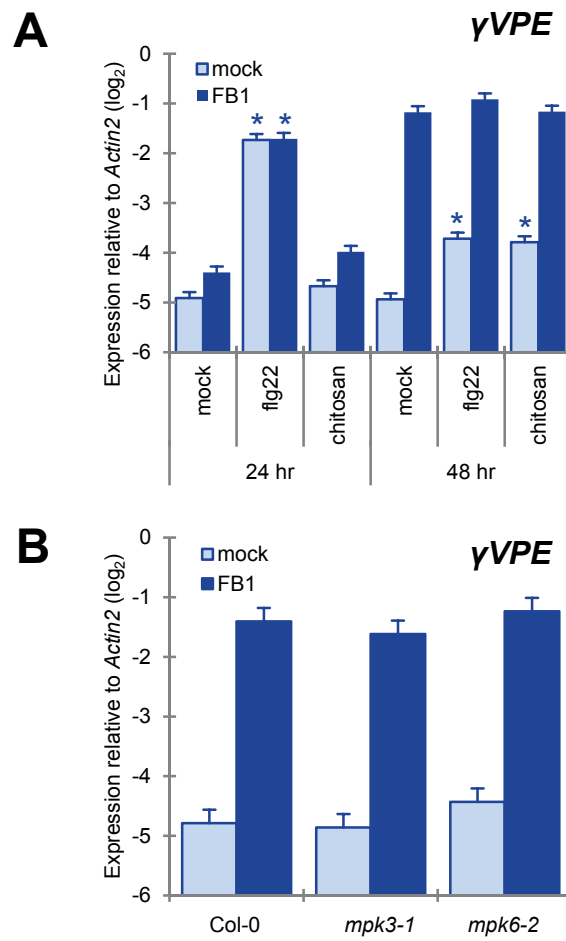

Supplement: Figure S5 — Induction of γVPE mRNA by FB1. The fifth and sixth true leaves of three-week old plants were treated with water (mock), 1 µM flg22 or 100 µg/ml chitosan (chitin) and 1% methanol (mock) or 50 µM FB1, and samples were collected at 24 and 48 hours after treatment. Each sample was a pool of four leaves, from two plants each. The mRNA levels of γVPE were measured by qRT-PCR. Bars represent means and standard errors for biological replicates, calculated using a mixed linear model: two biological replicates were performed for each of two independent experiments. The vertical axis depicts the log2 transformed expression values relative to that of Actin2 (At3g18780). Asterisks indicate significant differences between mock and MAMPs (A) or Col-0 and mpk mutants (B) (P<0.001, two-tailed t-tests). (PDF) [file pone.0060769.s005.pdf]
