# Supplementary material for: Pattern-Triggered Immunity Suppresses Programmed Cell Death Triggered by Fumonisin B1
Source: PLoS One. 2013 Apr 1;8(4):e60769. doi: 10.1371/journal.pone.0060769 (PMC3613394; doi:10.1371/journal.pone.0060769)
Supplement: Figure S4 — Quantification of FB1-triggered cell death by electrolyte leakage in mpk6-3 . This experiment was performed as described in Figures 4C and D. Bars represent means and standard errors for biological replicates, calculated using a mixed linear model: five to six biological replicates were performed for each of two independent experiments. (PDF) [file pone.0060769.s004.pdf]

Supporting Information Figure S4

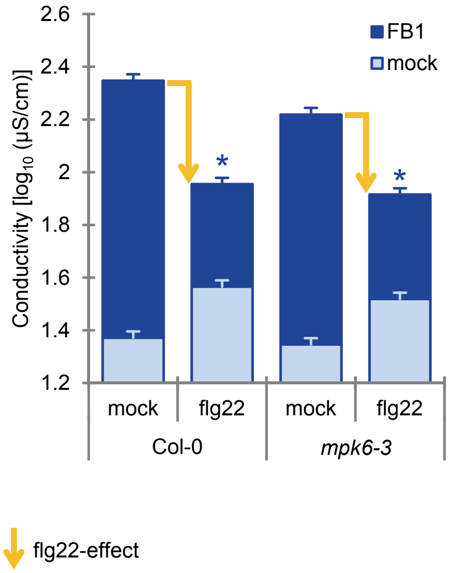

treatment	genotype	flg22 effect	*P*-value
FB1	Col-0	0.41	---
	mpk6-3	0.28	5.5×10^{-3}
